# Diagnosis of Early Alzheimer's Disease: Clinical Practice in 2021

**DOI:** 10.14283/jpad.2021.23

**Published:** 2021-06-09

**Authors:** A.P. Porsteinsson, R.S. Isaacson, Sean Knox, M.N. Sabbagh, I. Rubino

**Affiliations:** 1 University of Rochester School of Medicine and Dentistry, Rochester, NY, USA; 2 Weill Cornell Medical Center and New York-Presbyterian, New York, NY, USA; 3 Biogen International GmbH, Neuhofstrasse 30, 6340, Baar, Switzerland; 4 Cleveland Clinic Lou Ruvo Center for Brain Health s, Las Vegas, NV, USA; 5 Biogen Inc, Cambridge, MA, USA

**Keywords:** Alzheimer's disease, early diagnosis, diagnostic work-up

## Abstract

Alzheimer's disease is a progressive, irreversible neurodegenerative disease impacting cognition, function, and behavior. Alzheimer's disease progresses along a continuum from preclinical disease, to mild cognitive and/or behavioral impairment and then Alzheimer's disease dementia. Recently, clinicians have been encouraged to diagnose Alzheimer's earlier, before patients have progressed to Alzheimer's disease dementia. The early and accurate detection of Alzheimer's disease-associated symptoms and underlying disease pathology by clinicians is fundamental for the screening, diagnosis, and subsequent management of Alzheimer's disease patients. It also enables patients and their caregivers to plan for the future and make appropriate lifestyle changes that could help maintain their quality of life for longer. Unfortunately, detecting early-stage Alzheimer's disease in clinical practice can be challenging and is hindered by several barriers including constraints on clinicians' time, difficulty accurately diagnosing Alzheimer's pathology, and that patients and healthcare providers often dismiss symptoms as part of the normal aging process. As the prevalence of this disease continues to grow, the current model for Alzheimer's disease diagnosis and patient management will need to evolve to integrate care across clinical disciplines and the disease continuum, beginning with primary care. This review summarizes the importance of establishing an early diagnosis of Alzheimer's disease, related practical ‘how-to' guidance and considerations, and tools that can be used by healthcare providers throughout the diagnostic journey.

## Introduction

**D**ementia is among the greatest global health crises of the 21st century. Currently, more than 50 million people are living with dementia worldwide (1), with this number estimated to triple to 152 million by 2050 as the world's population grows older (2). Alzheimer's disease (AD) is the most common cause of dementia and is thought to account for 60–80% of dementia cases (3). Currently, the total annual cost for AD and other dementias in the USA is $305 billion and is predicted to increase to more than $1.1 trillion by 2050 (3). This substantial economic burden includes not only healthcare and hospice support for patients with AD (3) but also lost productivity from patients and caregivers (4).

AD is a progressive, neurodegenerative disease associated with cognitive, functional, and behavioral impairments, and characterized by two underlying pathological hallmarks: the progressive accumulation of extracellular amyloid beta (Aβ) plaques and intracellular neurofibrillary tangles (NFTs) (3). In AD, aggregated Aβ plaques are deposited within the brain as a result of either reduced Aβ clearance or excessive production (5); plaque deposition typically occurs ∼20 years before the onset of cognitive impairment (6, 7). NFTs are formed by the abnormal accumulation of hyperphosphorylated-tau protein (5); these can be detected 10–15 years before the onset of symptoms (6, 7).

**Figure 1:**
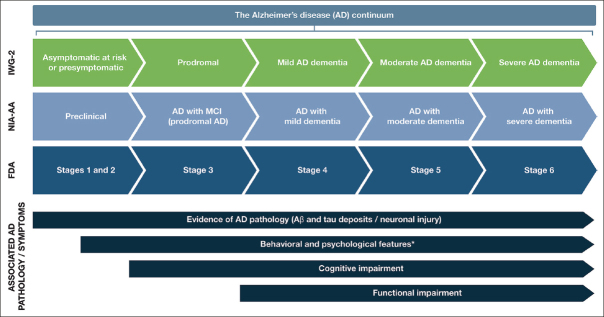
Stages within the Alzheimer's disease continuum

AD follows a progressive disease continuum that extends from an asymptomatic phase with biomarker evidence of AD (preclinical AD), through minor cognitive (mild cognitive impairment [MCI]) and/or neurobehavioral (mild behavioral impairment [MBI]) changes to, ultimately, AD dementia. A number of staging systems have been developed to categorize AD across this continuum (7, 8, 9). While these systems vary in terms of how each stage is defined, all encompass the presence/absence of pathologic Aβ and NFTs, as well as deficits in cognition, function, and behavior (7, 8, 9). As a result, subtle but important differences exist in the nomenclature for each stage of AD depending on the selected clinical and research classifications (Figure 1).

The AD continuum can be classified into different stages from preclinical AD to severe AD dementia; the nomenclature associated with each stage varies between the different clinical and research classifications. This figure provides a summary of the different naming conventions that are used within the AD community and the symptoms associated with each stage of the continuum; *Mild behavioral impairment is a construct that describes the emergence of sustained and impactful neuropsychiatric symptoms that may occur in patients ≥50 years old prior to cognitive decline and dementia (112); Abbreviations: Aβ, amyloid beta. AD, Alzheimer's disease. FDA, Food and Drug Administration. IWG, International Working Group. MCI, mild cognitive impairment. NIA-AA, National Institute on Aging—Alzheimer's Association

Preclinical AD, as the earliest stage in the AD continuum, comprises a long asymptomatic phase, in which individuals have evidence of AD pathology but no evidence of cognitive or functional decline, and their daily life is unaffected (8) (Figure 1). The duration of preclinical AD can vary between individuals, but typically lasts 6–10 years depending on the age of onset (10, 11). The risk of progression from preclinical AD to MCI due to AD (with/without MBI) depends on a number of factors, including age, sex, and apolipoprotein E (ApoE) status (11, 12); however, not all individuals who have underlying AD pathology will go on to develop MCI or AD dementia (13, 14). A recent meta-analysis of six longitudinal cohorts followed up for an average of 3.8 years found that 20% of patients with preclinical AD progressed to MCI due to AD (11). A further study by Cho et al., with an average follow-up rate of 4 years, found that 29.1% of patients with preclinical AD progressed to MCI due to AD (12).

For patients who do progress to MCI due to AD (with/without MBI), initial clinical symptoms typically include short-term memory impairment, followed by subsequent decline in additional cognitive domains (15) (Figure 1). On a day-to-day basis, an individual with MCI due to AD may struggle to find the right word (language), forget recent conversations (episodic memory), struggle with completing familiar tasks (executive function), or get lost in familiar surroundings (visuospatial function) (15, 16). As individuals have varying coping mechanisms and levels of cognitive reserve, patients' experiences and symptomology vary widely; however, patients tend to remain relatively independent at this stage, despite potential marginal deficits in function. The prognosis for patients with MCI due to AD can be uncertain; one study that followed up patients with MCI due to AD for an average of 4 years found that 43.4% progressed to AD dementia (12). Other studies reported 32.7% and 70.0% of individuals with MCI due to AD progress to AD dementia within 3.2 and 3.6 years of follow-up, respectively (17, 18). Patients who do progress to AD dementia will develop severe cognitive deficits that interfere with social functioning and will require assistance with activities of daily living (7) (Figure 1). As the disease progresses further, increasingly severe behavioral symptoms will develop that significantly burden patients and their caregivers, and the disease ultimately results in severe loss of independence and the need for round-the-clock care (3).

An early diagnosis of AD can provide patients the opportunity to collaborate in the development of advanced care plans with their family, caregivers, clinicians, and other members of the wider support team. Importantly, it also enables patients to seek early intervention with symptomatic treatment, lifestyle changes to maintain quality of life, and risk-reduction strategies that can provide clinically meaningful reductions in cognitive, functional, and behavioral decline (19, 20, 21, 22). It can also help reduce healthcare system costs and constraints: a study by the Alzheimer's Association found that diagnosing AD in the early stages could save approximately $7 trillion. These savings were due to lower medical and long-term care costs for patients with managed MCI than for those with unmanaged MCI and dementia (3). Furthermore, an early diagnosis will be vital for patients when a therapy addressing the underlying pathology of AD becomes available; currently 19 biologic compounds are under Phase 2 or 3 investigation (23). Physicians will need to be prepared for the approval of these treatments, to optimize the potential benefit and prolong preservation of patients' cognitive function and independence beyond that associated with current standard of care (19).

As the prevalence of AD continues to grow, the advancement of AD patient diagnosis will require an orchestrated effort, starting in the primary care setting and subsequently involving multiple healthcare provider (HCP) specialties (e.g., nurse practitioner [NP] or physician assistant [PA]) throughout the disease continuum. Galvin et al. recently highlighted the need for HCPs to work as an integrated, patient-centered care team to accommodate the growing and diverse population of patients with AD, beginning with diagnosis (24). For patients to receive a timely diagnosis, it is vital to implement an approach that minimizes the burden placed on the patient, clinician, and healthcare system (25). Here, we summarize the importance of establishing an early diagnosis of AD, related practical ‘how-to' guidance and considerations, and tools that can be used by healthcare providers throughout the diagnostic journey.

## The importance of an early diagnosis

Historically, a diagnosis of AD has been one of exclusion, and one only made in the latter stages of disease (26); however, the disease process can take years to play out, exacting a significant toll on the patient, caregiver, and healthcare system along the way (27).

To mitigate this burden, the early and accurate detection of AD-associated symptoms in clinical practice represents a critically needed but challenging advancement in AD care (19, 28, 29, 30). Usually, a patient with early signs/symptoms of AD will initially present in a primary care setting (30). For some patients, minor changes in cognition and/or behavior may be detected during a routine wellness visit or an appointment to discuss other comorbidities (24). As the PCP is often the first to observe a patient's initial symptomatology, it is vital they recognize the early signs and symptoms, and understand how to use the most appropriate assessment tools designed to detect these early clinical effects of the disease.

Because the neuropathologic hallmarks of AD (Aβ plaques and NFTs) can be detected decades prior to the onset of symptoms (6, 7), biomarkers reflecting this underlying pathology represent an important opportunity for early identification of patients at greatest risk of developing MCI due to AD. Biomarkers support the diagnosis of AD (especially important early on when symptoms can be subtle), and the U.S. Food and Drug Administration (FDA) has recently published guidelines that endorse their use in this population (9). The National Institute on Aging—Alzheimer's Association (NIA-AA) has recently created a research framework that acknowledges the use of biomarkers for diagnosing AD *in vivo* and monitoring disease progression (7).

Important biomarker information can be gathered from imaging modalities such as magnetic resonance imaging (MRI) and positive emission tomography (PET) that visualize early structural and molecular changes in the brain, respectively (25, 30). Fluid biomarker testing, such as cerebrospinal fluid (CSF) can also be used; CSF biomarkers can directly reflect the presence of Aβ and aggregated tau within the brain (7, 31). As will be discussed in more depth later in this article, a large number of clinical studies have shown that Aβ and tau biomarkers can contribute diagnostically important information in the early stages of disease (32). There is ongoing research to expand the current range of tests that can be used by clinicians as part of the multistage diagnostic process (25). For instance, once approved, blood-based biomarkers could be used to identify patients at risk of developing AD and for monitoring disease progression (33, 34), which would also reduce the current capacity constraints associated with PET imaging (25).

## Practical guide for an early diagnosis of Alzheimer's disease in clinical practice

**Figure 2:**
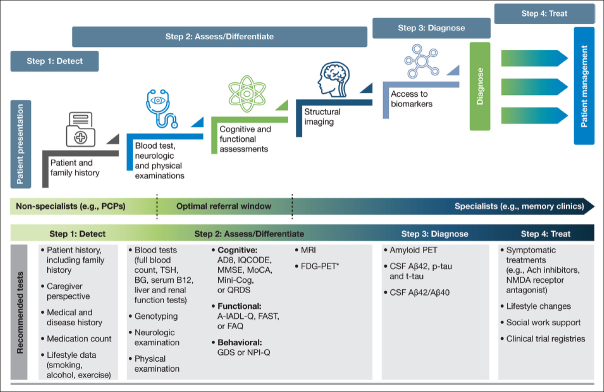
A stepwise infographic to highlight key stages within the diagnostic process, along with the recommended tests to support each step

As already raised, recent recommendations for evolving AD care to a more patient-centric, transdisciplinary model include guidance on realizing an efficient diagnostic process—one in which HCPs, payers, and specialists are encouraged to combine their efforts to ensure the early warning signs of AD are not overlooked (24). The recommendations include dividing the diagnosis of AD into the following steps: detect, assess/differentiate, diagnose, and treat (Figure 2). We present here a practical guide for the early diagnosis of AD, based on this outlined approach, including a case study to highlight each of these key steps.

The diagnostic process for AD can be divided into the following steps: detect, assess/differentiate, diagnose, and treat. It is important for clinicians to utilize appropriate tests when investigating a patient suspected of having AD in the early stages. Here, we highlight the most valuable tests for each step and which ones should be used in a primary care or specialist setting; *FDG-PET is usually considered after a diagnostic work-up; Abbreviations: A-IADL-Q, Amsterdam Instrumental Activities of Daily Living Questionnaire. Aβ, amyloid beta. Ach, acetylcholine. BG, blood glucose. CSF, cerebrospinal fluid. FAQ, Functional Activities Questionnaire. FAST, Functional Analysis Screening Tool. FDG-PET, fluorodeoxyglucose-PET. GDS, Geriatric Depression Scale. IQCODE, Informant Questionnaire on Cognitive Decline in the Elderly. Mini-Cog, Mini Cognitive Assessment Instrument. MMSE, Mini-Mental State Examination. MoCA, Montreal Cognitive Assessment. MRI, magnetic resonance imaging. NMDA, N-Methyl-D-aspartic acid. NPI-Q, Neuropsychiatric Inventory Questionnaire. PCP, primary care physician. PET, positive emission tomography. p-tau, phosphorylated tau. QDRS, Quick Dementia Rating System. TSH, thyroid-stimulating hormone. t-tau, total tau depressive symptoms and anxiety, as well as irritability. Based on the patient's symptoms, the PCP felt his presentation warranted further clinical assessment.

## Step 1: Detect

### The role of primary care in the early detection of AD

The insidious and variable emergence of symptoms associated with AD and other dementias can make recognition extremely challenging, particularly in a primary care setting (30, 35). Clinicians often have limited time with patients, so it is vital that they are able to quickly and accurately recognize the early signs and symptoms associated with AD (Table 2) (3, 30, 36), and training for nurses, NPs, and PAs who may have more time to observe patients should provide substantial benefits. Although extremely variable, initial symptoms may include short-term memory loss or psychological concerns, including depressive symptoms and a loss of purpose (36).

**Table 2:** Symptoms associated with suspected early stage Alzheimer's disease

Area	Cognition (3, 30)	Behavior (3, 30, 36)	Psychological (3, 36)	Physical (3, 36	Other (36)
**Category**	• Short-term memory loss • Word-finding difficulties (anomia) or communication difficulties	• Withdrawal from social activities • Disinhibition and impulsivity	• Depression • Mood disturbances • Apathy	• Visuospatial problems • Gait impairment	• Sleep disorder
**Examples**	• Forgetting appointments, names, and recent events • Frequently misplacing items • Trouble finding exact words to express oneself, or loss of word meaning	• Inability to participate in meaningful social situations • Inappropriate social conduct such as eating from someone else's plate, or inappropriate language • Poor or decreased judgment	• Changes in mood or personality • Feeling of helplessness and a loss of purpose in life • Loss of initiation	• Frequent falls	• Rapid eye movement disorder, such as acting out dreams

Patients, family members, and even HCPs themselves may present barriers to the diagnosis of early-stage AD. Patients may hide their symptoms or even avoid making an appointment until their symptoms significantly affect their day-to-day life due to fear of the stigma associated with a diagnosis of AD (19). Additionally, patients, family members, and PCPs/HCPs may dismiss or misinterpret symptoms as simply part of the normal aging process (30). Retrieving information from a trusted family member or informant/caregiver is essential when trying to assess a patient for suspected AD, as this perspective can provide a more objective understanding of the daily routine, mood, and behavior of the patient, and how this may have changed over time (30). For patients presenting with even subtle symptoms associated with AD, it is important that the PCP/HCP conducts an initial assessment to confirm the presence of symptoms using a validated assessment for early-stage AD detection (Figure 2; Step 2: Assess/Differentiate).

### Case study: Presentation

A 63-year-old Caucasian male (J.K.) presented to his PCP with short-term memory loss over the last 2 years (Table 1A). Accompanied by his wife, he acknowledged his job had been affected by issues with his short-term memory; however, he considered his memory similar to that of his peers. His wife reported that people at work had started to notice him struggling to keep up, and also that family had to remind him of his upcoming appointments. He admitted to having intermittent

**Table 1:** Patient case study

A - Presentation	
• A 63-year-old Caucasian male patient (J.K) visited the memory clinic accompanied by his wife, having been referred by his PCP for evaluation of memory loss	
• He presents with a history of an insidious onset of cognitive difficulties that have been progressive over the past 2 years. He considers his memory similar to his peers, and his deficits are not observable to people who know him casually	
• At work, he has uncharacteristically confused orders and misplaced items, but has no difficulty keeping track of time, and his math, reading, and writing are intact. His wife says that people at work have started to notice him struggling to keep up and gently voiced their concerns to her	
• The patient's basic activities of daily living are intact, but more complex instrumental activities of daily living are showing erosion. He still drives, but no longer wants to drive to areas he is not familiar with	
• He presents with no gait difficulty or balance problems. In terms of neuropsychiatric symptoms, his mood is more labile. He chokes up easily and is overall a little more down but attributes this to the fear and frustration over what is happening to him. He does have some mixed neuropsychiatric symptoms with intermittent depressive symptoms and anxiety as well as irritability	
• Past medical history significant for hypertension, dyslipidemia, mild obesity, and glucose intolerance	
• No history of neurotoxic exposure, head injuries with post-concussion syndrome, strokes, or seizures	
• A positive family history of dementia with his father and paternal grandmother, where onset occurred in the late 60s	
**C - Assess/Differentiate**	
**Blood tests**:	All normal, except for serum glucose of 115 and HgbA1c of 6.5%
**Neurologic examination**:	Non-focal with faint bilateral palmomental reflex
**Genotyping**:	Homozygous for ApoE ε4; no autosomal dominant genes
**Cognitive assessments**:	MoCA score of 21/30
**Structural imaging**:	MRI showed mild small vessel disease and mild generalized atrophy Hippocampal volume and ratio were reduced by 25% based on volumetric software
**D - Diagnose**	
**CSF biomarkers**:	Increased p-tau and t-tau Reduced Aβ42 Aβ42/40 ratio of 0.23
**Diagnosis**:	The most likely etiology is Alzheimer's disease, especially in view of a positive family history with similar age of onset, ApoE ε4 status, and biomarker verification
**E - Treat**	
• Advised patient to make lifestyle modifications, including controlling vascular risk factors and optimizing the management of other medical problems	
• No treatment intervention required for neuropsychiatric symptoms at the time of diagnosis	
• Provided information on local social worker to help support him and his family	
• Encouraged regular follow-ups and monitoring	
• Patient was referred for possible participation in a clinical trial	


Abbreviations: Aβ, amyloid beta. ApoE, apolipoprotein E. HgbA1c, hemoglobin A1c. MoCA, Montreal Cognitive Assessment. MRI, magnetic resonance imaging. PCP, primary care physician. p-tau, phosphorylated tau. t-tau, total tau

## Step 2: Assess and differentiate

### Primary care: Initial assessment when a patient presents

When a patient initially presents with symptoms consistent with early stages of AD, a clinician must first conduct a comprehensive clinical assessment to rule out other potential non-AD causes of cognitive impairment (Figure 2). PCPs are well placed to conduct these initial assessments, as they may not require specialist input or hospital tests. During the initial assessment, the primary objective of the clinician should be to exclude possible reversible causes of cognitive impairment, such as depression, or vitamin, hormone, and electrolyte deficiencies (37). The initial assessment should include a thorough history to identify potential risk factors associated with AD, including a family history of AD or related dementias in first-degree relatives (31, 38). Other known risk factors for AD that should be identified include age, female sex, ApoE ε4 status, physical inactivity, low education, diabetes, and obesity (3). It is also important to review for pre-existing medical conditions or prescribed medications that could be a cause of the patient's cognitive impairment (36). Additionally, when conducting a thorough history, open-ended, probing questions should be directed to both the patient and the informant to ascertain how the patient's cognition has changed over time and how the cognitive deficits affect their everyday activities; example questions for the initial assessment are detailed in Table 3 (30). Engaging with informants/caregivers is key to capturing additional information to help support all assessments. A routine differential diagnosis of AD begins with a detailed history, physical and neurologic examinations, and bloodwork analyses, followed by cognitive assessments and functional evaluation (Figure 2).

**Table 3:** Example questions for a clinician conducting an initial assessment with a patient and caregiver (30)

Required information	Example questions for the patient and/or informant
Medical history	Has the patient had any recent illnesses? Has the patient recently had any head injuries? Has the patient used any medications recently that could cause memory loss? Has the patient used or been exposed to any illicit drugs? Is there a history of epilepsy?
Risk factors	Is there a history of dementia within the family?
	Does the patient have any other medical conditions, such as cardiovascular disease or obesity?
	Is the patient a smoker or ex-smoker?
Cognitive and behavioral changes	What does a typical day look like for you (the patient)? Has the patient noticed they are forgetting things or misplacing items recently? Has the patient noticed any changes to their mood or felt helpless recently? Has the patient had any issues with finances?
Physical	Has the patient had any falls recently? Has the patient noticed any issues with their balance?
Other	Does the patient have any vision or hearing problems? Is there anything else the patient or caregiver is concerned about?

### Primary care: Physical examination and blood analyses

A physical examination and blood tests can identify comorbid contributory medical conditions and reversible causes of cognitive impairment. A physical examination, including a mental status and neurological assessment, should be conducted to detect conditions such as depression and, for example, to look for signs such as issues with speaking or hearing as well as signs that could indicate a stroke (37). As part of the physical exam, a physician may ask the patient about diet and nutrition, review all medications (to see if these are the cause of any cognitive impairment, e.g. anti-cholinergics, analgesics, or sleep aids and anxiolytics), check blood pressure, temperature and pulse, and listen to the heart and lungs (36, 39).

Blood tests can rule out potentially treatable illnesses as a cause of cognitive impairment, such as vitamin B_12_ deficiency or thyroid disease (37). Suggested blood analyses include: 1) complete blood cell count; 2) blood glucose; 3) thyroid-stimulating hormone; 4) serum B_12_ and folate; 5) serum electrolytes; 6) liver function; and 7) renal function tests (30). Although not routinely used in clinical practice, clinicians may request ApoE genotyping, as this can help assess the genetic risk of developing AD. ApoE is the dominant cholesterol carrier within the brain that supports lipid transport and injury repair (40, 41), and the APOE gene exists as three polymorphic alleles: APOE ε2, ε3, and ε4. The ε4 allele of ApoE is associated with increased AD risk, whereas the ε2 allele is protective (40, 42). The number of ApoE ε4 alleles a person carries increases their risk of developing AD and the age of disease onset (43). Homozygous ε4 carriers (those with two copies of the ε4 allele) have the greatest risk of developing AD and the lowest average age of onset (43). In some practice settings, ApoE genotyping can only be conducted by a genetic counselor; a referral for more comprehensive genetic testing may be considered by the HCP if there is a family history of early-onset AD or dementia. Consumer tests are also becoming more readily available for patients wanting to determine their risk of developing diseases such as AD based on genetic risk factors (44).

### Primary care: Cognitive, functional, and behavioral assessments

#### Cognitive assessments

If a patient is suspected of having AD following an initial assessment in primary care, and they are <65 years old, or if the case is complex, a referral to a dementia specialist such as a neurologist, geriatrician, or geriatric psychiatrist may be required for further evaluation. The specialist would then use an appropriate battery of cognitive, functional, and behavioral tests to assess the different aspects of disease, and ultimately to confirm diagnosis. However, not all patients with suspected cognitive deficits are immediately referred to a dementia specialist at this stage, which is only partly due to limited numbers of specialists (25) (Figure 2). In clinical practice, a two-stage process is often employed. This involves an initial ‘triage' step conducted by non-specialists to clinically assess and select those patients who require further evaluation by a dementia specialist (45). During this ‘triage' step, there are several clinical assessments available to non-specialists for assessing the presence of cognitive and functional impairments and behavioral symptoms (Table 4) (28, 35, 46, 47, 48, 49, 50, 51, 52, 53, 54, 55).

**Table 4:** Cognitive, functional, and behavioral assessments to support the diagnosis of Alzheimer's disease in a primary care and specialist setting

Primary Care									
**Type of assessment**	**Assessment**	**Number of items (if appropriate)**	**Time taken to complete assessment (minutes)**	**Scoring system**	**Sensitivity and specificity**	**Available in different languages**	**Shortened version available**	**Scores can be demographically adjusted, e.g. education level**	**Justification for use**
Cognitive	MMSE (28, 102, 103)	30	5–10	23–24	Sensitivity: 85–100% Specificity: 66–100%	Yes	Yes	Yes	Minimal training requirements
	MoCA (28, 46, 102, 104)	12	10	<26 for MCI or dementia	Sensitivity: 78–100% Specificity: 65–94%	Yes	Yes	Yes	Minimal training requirements
	Mini-Cog (28, 102, 105)	3 item recall with clock drawing	2–3	Recall 2/3 items Clock drawing used to determine presence of cognitive deficits	Sensitivity and specificity comparable to MMSE	Yes	No	No	Brief assessment and easy to interpret No training requirements
	AD8 (28, 106	8	2–3	Scores greater than 2 signify impairment	Sensitivity: 90% Specificity: 68%	Yes	No	No	Brief assessment for cognitive impairment
	IQCODE (28, 107	16 or 26	10	Scores greater than 3.44 signify impairment	Sensitivity: 76–100% Specificity: 65–86%	Yes	Yes	No	Measures decline from premorbid level
Functional	FAQ (46^8,^108^)	10 categories	5*	0–3 scale (0=normal; 3=dependent)	Sensitivity: 90% Specificity: 83%	Yes	No	No	Highly reliable assessment
Behavioral	GDS (28, 49, 109)	15 or 30	5–10	≥5 suggestive of depression; ≥10 significant of depression*	No data available	Yes	Yes	No	Reliable assessment for early stages of dementia
	NPI-Q (49, 50, 51, 110)	12	5	0-3 scale (0=none; 3=severe)	Sensitivity: 86% Specificity: 76%	Yes	No	No	Brief and reliable assessment
**Specialist**									
**Type of assessment**	**Assessment**	**Number of items (if appropriate)**	**Time taken to complete assessment (minutes)**	**Scoring system**	**Sensitivity and specificity**	**Available in different languages**	**Shortenedversionavailable**	**Scores can be demographically adjusted, e.g. education level**	**Justification for use**
Cognitive	QDRS (28, 52	10	3–5	Scores of 2 or greater signify impairment	Sensitivity: 84% Specificity: 75%	Yes	No	No	Highly reliable assessment. No training requirements
Functional	A-IADL-Q (53, 54, 111)	≤70 items	10	5-point rating system	Sensitivity: 74% Specificity: 65%	Yes	Yes	No	Sensitive to early stages of AD
	FAST (55)	28	10–15	Yes/No for presence of behavioral concern	No data available	No	No	No	Useful test to assess behavioral concerns from multiple informants


*Personal communication; Abbreviations: AD, Alzheimer's disease. A-IADL-Q, Amsterdam Instrumental Activities of Daily Living Questionnaire. FAQ, Functional Activities Questionnaire. FAST, Functional Assessment Screening Tool. GDS, Geriatric Depression Scale. IQCODE, Informant Questionnaire on Cognitive Decline in the Elderly. MCI, mild cognitive impairment. Mini-Cog, Mini Cognitive Assessment Instrument. MMSE, Mini-Mental State Examination. MoCA, Montreal Cognitive Assessment. NPI-Q, Neuropsychiatry Inventory Questionnaire. QDRS, Quick Dementia Rating System

Previous research has shown that clinicians have a tendency to choose one assessment over another due to their familiarity with the assessment, time constraints, or specific resources available to them within their clinic (30), but clinicians need to be aware of, and prepared to use, the most patient-appropriate assessments: the cultural, educational, and linguistic needs of the patient are important considerations (30, 36, 56, 57, 58). Some assessments have been translated into different languages or shortened, or have education-adjusted scoring classifications, where required (56, 57, 58).

Cognitive assessments that can be conducted quickly (<10 minutes), such as the Mini-Mental State Examination (MMSE) or Montreal Cognitive Assessment (MoCA), can be used by non-specialists to identify the presence and severity of cognitive impairment in patients before referring to a dementia specialist (Table 4) (36). Both the MMSE and MoCA are used globally in clinical practice, particularly in primary care, but vary in terms of their sensitivity to identify AD in the early stages (28, 59). The MMSE is sensitive and reliable for identifying memory and language deficits in general but has limitations in identifying impairments in executive functioning (59). MoCA was originally developed to improve the detection of MCI (28) and is more sensitive than the MMSE in its assessment of memory, visuospatial, executive, and language function, and orientation to time and place (59). Both tests are relatively easy to administer and take around 10 minutes to complete. Neither assessment requires extensive training by the clinician, although MoCA users do need to undergo a 1-hour certification as mandated by the MoCA Clinic and Institute (28, 60).

For time-constrained clinicians, the Mini Cognitive Assessment Instrument (Mini-Cog) may be an appropriate tool to assess cognitive deficits that focus on memory, and components of visuospatial and executive function (Table 4). The assessment includes the individual learning three items from a list, drawing a clock, and then recalling the three-item list. The Mini-Cog can be useful for clinicians in primary care, as it requires no training and the results are easy to interpret. As an alternative to these tests, PCPs might also consider using an informant-based structured questionnaire such as the AD8 or Informant Questionnaire on Cognitive Decline in the Elderly to help guide discussions with the patient and caregiver (Table 4) (28).

#### Functional assessments

Functional assessments are valuable in identifying changes in a patient's day-to-day functioning through the evaluation of their instrumental activities of daily living (IADLs). IADLs are complex activities that are necessary for the individual to function independently (e.g., cooking, shopping, and managing finances) and can be impaired during the early stages of cognitive impairment. While it is possible that functional decline may occur as a part of normal aging, a decline in a person's IADL performance is strongly associated with neurodegenerative diseases such as AD (61). In the early stages of AD, patients may be functionally independent, and any impairment in IADLs may be subtle, such as difficulties paying bills or driving to new places. A patient's functional independence is essential for their well-being and mental health (62), particularly in the early stages of the disease when the individual may still be working and socializing relatively independently (3). Consequently, functional independence is one of the most important clinical features for patients with AD. As the disease progresses, and patients have increasing functional impairment, this significantly impacts on their independence, and subsequently their and their family/caregiver's quality of life.

Functional assessment is, therefore, an integral part of the diagnostic process for AD. The Functional Activities Questionnaire (FAQ) is an informant questionnaire that assesses the patient's performance over a 4-week period and may take only a few minutes to complete (Table 4). The questionnaire is scored from ‘normal' to ‘dependent', using numerical values assigned to categories, with higher scores indicative of increasing impairment (47). Previous research has shown that the FAQ has high sensitivity and reliability for detecting mild functional impairment in patients with MCI (47).

Determining an individual's functional independence can be challenging and the clinician may require additional input from an informant to determine a patient's functional decline and their ongoing ability to conduct activities of daily living (37). The clinician can gain greater insight through the informant into the patient's day-to-day life and any issues the patient is having at home. This type of information is vital to the clinician, and when combined with other assessment tools, can help to narrow the differential diagnosis.

#### Behavioral assessments

Patients with suspected AD may experience several behavioral symptoms such as anxiety, disinhibition, apathy, and depression (Table 2). In the early stages of disease, such symptoms are generally associated with poor long-term outcomes and caregiver burden, and are particularly distressing to both patients and their families (63). It is important for clinicians to use appropriate assessments to identify behavioral and psychiatric symptoms that are caused by neurodegenerative diseases, such as AD, rather than by alternative causes, such as a mood disorder.

The Geriatric Depression Scale (GDS) and Neuropsychiatric Inventory Questionnaire (NPI-Q) can be used by clinicians to assess neuropsychiatric symptoms in patients for whom early-stage AD is suspected (Table 4). The GDS is a 15-item (or longer 30-item) questionnaire that assesses mood, has good reliability in older populations for detecting depression, and can be completed by the patient within 5–10 minutes (63). The NPI-Q can be used in conjunction with or as an alternative to the GDS. The NPI-Q is completed by a knowledgeable informant or caregiver who can report on the patient's neuropsychiatric symptoms. The NPI-Q can be conducted in around 5 minutes to determine both the presence and severity of symptoms across several neuropsychiatric domains including depression, apathy, irritability, and disinhibition (49). Consequently, as it assesses depression, it can be used as an alternative to GDS if time constraints do not allow for both to be completed.

Behavioral symptoms can be non-specific, so it is important for clinicians to consider and rule out other potentially treatable causes of impairment when assessing this domain. For example, depression is associated with concentration and memory issues (64); apathy can occur in non-depressed elderly individuals and can impact cognitive function (65). Signs/symptoms such as social withdrawal, feelings of helplessness, or loss of purpose should be investigated closely, as these could be indicative of depression alone. It is important for clinicians to recognize that if changes over time in cognitive symptoms and mood symptoms match, then depression is most likely to be the root cause of subtle cognitive decline, rather than AD (28).

### Primary care clinician checklist

If AD is still suspected following clinical assessment, referral to a specialist for further diagnostic testing, including imaging and fluid biomarkers, may be required. It is important the clinician confirms the following checks/assessments before the patient undergoes further evaluation:

### Primary care clinician checklist

• Confirm medical and family history
• Review the patient's medications for any that could cause cognitive impairment
• Perform blood tests to eliminate potential reversible causes of cognitive impairment
• Conduct a quick clinical assessment to confirm the presence of cognitive impairment

### Specialist role in assessment

Following the initial assessment in primary care, further cognitive, behavioral, functional, and imaging assessments can be carried out in a specialist setting. With their additional AD experience, access to other specialties, and possibly fewer time constraints than the PCP, the specialist is able to conduct a more comprehensive testing battery, using additional clinical assessments and biomarkers to determine causes of impairment and confirm diagnosis (Figure 2).

#### Cognitive assessments

Because the cognitive impacts of early-stage AD may vary from patient to patient, it is important to consider which cognitive domains are affected in these early stages when considering which assessments to use. Specialists are able to conduct a full neuropsychological test battery that covers the major cognitive domains (executive function, social cognition/emotions, language, attention/concentration, visuospatial and motor function, learning and memory); preferably, a battery should contain more than one test per domain to ensure adequate sensitivity in capturing cognitive impairment (66). This step can help with obtaining an in-depth understanding of the subtle changes in cognition seen in the early stages of AD and enables the clinician to monitor subsequent changes over time.

Typically, episodic memory, executive function, visuospatial function, and language are the most affected cognitive domains in the early stages of AD (29, 67, 68). Currently, most cognitive assessment tools focus on a subset of the overall dimensions of cognition; it is therefore vital the clinician chooses the correct test to assess impairment in these specific cognitive domains that could be indicative of AD in the early stages. As cognitive impairment in the early stages of AD can be subtle and vary significantly between individuals (29), clinicians must choose appropriate, sensitive tests that can detect these changes and account for a patient's level of activity and cognitive reserve (29). If there is large disparity in results across cognitive assessments, it is important for the clinician to shape their assessments based on the patient's history. If the patient's history is positive for neurodegenerative disease, but one assessment does not reflect this, it is important to conduct further tests to ascertain the cause of the cognitive impairment.

The Quick Dementia Rating System (QDRS) can be used by specialists to assess cognitive impairment (Table 4). This short questionnaire (<5 minutes) is completed by a caregiver/informant and requires no training. The QDRS assesses several cognitive domains known to be affected by AD, including memory, language and communication abilities, and attention. The questionnaire can reliably discriminate between individuals with and without cognitive impairment and provides accurate staging for disease severity (28).

#### Functional assessments

The Amsterdam IADL Questionnaire (A-IADL-Q) and Functional Assessment Screening Tool (FAST) can both be used to assess a patient's functional ability (Table 4) (53). The A-IADL-Q is a reliable computerized questionnaire that monitors a patient's cognition, memory, and executive functioning over time. This questionnaire is completed by an informant of the patient and takes 10 minutes to complete (53). For patients with suspected early stage AD, the A-IADL-Q is a useful tool to monitor subtle changes in IADL independence over time and is less influenced by education, gender, and age than other functional assessments (53). The FAST is a useful assessment for clinicians to identify the occurrence of functional and behavioral problems in patients with suspected AD. The questionnaire is completed by informants who interact with the patient regularly; informants are required to answer Yes/No to a number of questions focusing on social and non-social scenarios (55).

### Structural imaging

Structural imaging, such as MRI, provides clinically useful information when investigating causes of cognitive impairment (69) (Figure 2). MRI is routinely conducted to exclude alternative causes of cognitive impairment, rather than support a diagnosis of AD (37, 70). It is well known that medial temporal lobe atrophy is the best MRI marker for identifying patients in the earliest stages of AD (70, 71); however, specific patterns of atrophy may also be indicative of other neurodegenerative diseases. Atrophy alone is rarely sufficient to make a diagnosis. MRI findings can help to narrow the differential diagnosis, and the results should be considered in the context of the patient's age and clinical examination (69, 70, 71).

Clinicians are advised to take a stepwise approach when reviewing structural imaging reports of a patient with suspected AD. These steps include: 1) excluding brain pathology that may be amenable to surgical intervention (e.g., the scan will show regions of hyper- or hypointensity rather than a uniform signal); 2) assessing for brain microbleeds (e.g., looking at signal changes within different areas of the brain can identify vascular comorbidities); and 3) assessing atrophy (e.g., medial temporal lobe atrophy is characteristic of AD) (69). Radiologists can conduct a quick and easy visual rating of any medial temporal lobe atrophy; these results can then be utilized by the specialist, in conjunction with a clinical assessment, to determine the likely cause of cognitive impairment. If the clinician is unable to determine a differential diagnosis, additional confirmatory tests can be requested.

Fluorodeoxyglucose-PET (FDG-PET) is a useful structural imaging biomarker that can support an early and differential diagnosis (72); however, specialists usually prefer to use this after their initial diagnostic work-up. As the brain relies almost exclusively on glucose as its source of energy, FDG (a glucose analog) can be combined with PET to identify regional patterns of reduced brain metabolism and neurodegeneration (70, 72). FDG-PET is not recommended for diagnosing patients with preclinical AD, as there is no way to ascertain whether the hypometabolism is directly related to AD pathology (73); however, clinicians may refer patients with more established symptomatology for an FDG-PET scan to identify regions of glucose hypometabolism and neurodegeneration that could be indicative of AD (70).

### Case study: Assess/differentiate

The initial assessment by the primary care clinician revealed that J.K.'s medical history was significant for hypertension, dyslipidemia, mild obesity, and glucose intolerance (Table 1B). There was no history of cerebrovascular events, significant head injuries, or focal findings on the neurologic exam. Besides the vascular risk factors, no medical conditions or current medications were found to be likely contributors to the cognitive deficit. The patient had a positive family history of dementia, where the onset typically occurred in the late 60s. Genotyping showed the patient to be a homozygous carrier of two ApoE ε4 alleles. Blood tests revealed elevated serum glucose and C-reactive protein but were otherwise normal. The patient had an unremarkable mental status examination, and his MoCA score was 21/30, with points lost on orientation, recall, and naming (Table 1C).

The patient was referred to a memory clinic for further assessment. The dementia specialist referred the patient for an MRI that predominantly showed mild small vessel disease and mild generalized atrophy with a significant reduction in hippocampal volume and ratio. Based on his medical and family history, cognitive assessments, and structural imaging results, the specialist deemed the severity of cognitive impairment to be in the mild range; consequently, the specialist referred the patient for biomarker assessment to determine the underlying cause.

## Step 3: Diagnose

Historically, AD was only diagnosed postmortem until we developed the ability to ascertain the underlying pathology associated with the disease in new ways, namely imaging and fluid biomarkers. However, despite supportive results from single- and multicenter trials, the use and reimbursement of imaging and fluid biomarkers to support the diagnosis of AD still vary considerably between countries (70).

### Imaging biomarkers

Recent advances have allowed physicians to visualize the proteins associated with AD, namely Aβ and tau, via PET scanning. Amyloid PET is currently the only imaging approach recommended by the Alzheimer's Association and the Amyloid Imaging Task Force to support the diagnosis of AD (70). Amyloid PET utilizes tracers (florbetapir, flutemetamol, and florbetaben) that specifically bind to Aβ within amyloid plaques; a positive amyloid PET scan will show increased cortical retention of the tracer in regions of Aβ deposition within the brain (74), thus confirming the presence of Aβ plaques in the brain (74, 75) and directly quantify brain amyloid pathology (76), thus making it a useful tool to supplement a clinical battery to diagnose AD (3, 74). However, a positive amyloid PET scan alone does not definitively diagnose clinical AD, and these results must be combined with other clinical assessments, such as cognitive assessment, for an accurate diagnosis (74). It is also important to note that amyloid PET is expensive and not readily reimbursed by health insurance providers (70); if it is not possible to access amyloid PET, biomarker confirmation can be assessed using CSF.

### Fluid biomarkers

An additional or alternative tool to amyloid PET is the collection and analysis of CSF for the presence of biomarkers associated with AD pathology. Patients who have symptoms suggestive of AD can be referred for a lumbar puncture to analyze their CSF for specific AD-associated biomarkers (3). CSF biomarkers are measures of the concentrations of proteins in CSF from the lumbar sac that reflect the rates of both protein production and clearance at a given timepoint (7). Lumbar punctures can be conducted safely and routinely in an outpatient setting or memory clinic (77). However, many patients still worry about the pain and possible side effects associated with the procedure and may require additional information and support from the clinician to undertake the procedure (77). Appropriate use criteria are available for HCPs to help identify suitable patients for lumbar puncture and CSF testing (78). For example, individuals presenting with persistent, progressing, and unexplained MCI, or those with symptoms suggestive of possible AD, should be referred for lumbar puncture and CSF testing (78). However, lumbar puncture and CSF testing are not recommended for determining disease severity in patients who have already received a diagnosis of AD or in lieu of genotyping for suspected autosomal dominant mutation carriers (78).

Because there is strong concordance between CSF biomarkers and amyloid PET, either can be used to confirm Aβ burden (79). As such, CSF biomarkers are widely accepted within the AD community to support a diagnosis (80). AD biomarkers from the brain can be detected in CSF well before the onset of overt clinical symptoms in early-stage AD (6, 7). Core AD CSF biomarkers, such as Aβ42 (one of two main isoforms of Aβ and a major constituent of Aβ plaques) and phosphorylated tau (p-tau) and total tau (t-tau), can be measured to determine the presence of disease (80).

When interpreting CSF analyses for a patient with suspected AD, it is important to remember that AD is associated with decreased CSF Aβ42 and increased tau isoforms (32). Decreased CSF Aβ42 levels are a reflection of increased Aβ aggregation and deposition within the brain (32), and the concentration of CSF Aβ42 directly relates to the patient's amyloid status (e.g., the presence or absence of significant amyloid pathology) and the total amount of Aβ peptides (e.g., Aβ42 and Aβ40) (32). Specialists' use of ratios of these CSF biomarkers (e.g., Aβ42/40) rather than single CSF biomarkers alone has been shown to adjust for potential differences in Aβ production and provide a better index of the patient's underlying amyloid-related pathology (81). The increase in CSF p-tau and t-tau associated with AD may directly reflect the aggregation of tau within the brain and neurodegeneration, respectively (32). P-tau in CSF provides a direct measure of the amount of hyperphosphorylated tau in the brain, which is strongly suggestive of the presence of NFTs, whereas CSF t-tau can predict the level of neurodegeneration in a patient with suspected AD; however, t-tau is also increased in other neurologic conditions (32).

Ultimately, the clinical decision to use amyloid PET or CSF to confirm amyloid and tau pathology can be affected by several practical factors (Table 5) (70, 77, 80, 82, 83, 84, 85).

**Table 5:** Comparison of key CSF and amyloid PET considerations for amyloid confirmation

Factor	Amyloid PET	CSF
Cost	High cost (70)	Moderate cost (82
Radiation	Yes (83	No
Headache	Very rare (80	Post-lumbar puncture headache (<2%) (80
Technique	Visual interpretation depends on the observer's experience (84	Reluctance around lumbar puncture (77
	Lacks a clear cutoff value between normal and pathologic findings (84	Patient assessed for coagulopathy; anticoagulant therapy contraindicated (85
		Complicated lumbosacral spinal anatomy (85
		Skin infection around puncture site (85


Abbreviations: CSF, cerebrospinal fluid. PET, positron emission tomography

### Emerging diagnostic tools

Access constraints for amyloid PET have driven the need for alternative sensitive and specific CSF and blood-based biomarkers that can detect AD-associated pathology in the early stages (86). Significant efforts have been undertaken over the last decade to identify blood-based biomarkers to: 1) detect AD pathology; 2) identify those at risk of developing AD in the future; and 3) monitor disease progression (33, 34, 87). At present, only a limited number of approved blood-based assays are available to clinicians to detect AD pathology (88); however, several novel assays are currently under investigation, including those measuring various phosphorylated forms of tau, including p-tau181 and p-tau217 (89). Investigational use of plasma p-tau181 (an isoform of tau) has been shown to differentiate AD from other neurodegenerative diseases and predict cognitive decline in patients with AD (33). CSF p-tau217 (a different isoform of tau) is a promising biomarker under investigation for detecting preclinical and advanced AD (86, 90). Given that blood testing is already a well-established part of clinical routines globally and can easily be performed in a variety of clinical settings, blood-based biomarkers could in future serve as the potential first step of a multistage diagnostic process. This would be a benefit to clinicians, particularly those in primary care, by helping to identify individuals requiring a referral to a specialist for diagnostic testing (87).

### Case study: Diagnose

J.K. underwent a lumbar puncture for CSF analysis, which showed decreased Aβ42 and increased p-tau and t-tau protein (Table 1D). Based on the results from the genotyping, cognitive assessments, MRI, and CSF biomarkers, the clinician confirmed that the likely cause of the patient's cognitive deficits was early-stage AD, especially in view of a positive family history of dementia with similar age of onset.

## Step 4: Treat

The role of the clinician following a diagnosis of early-stage AD is to discuss the available management and treatment options while providing emotional and practical support to the patient, caregiver, and family where appropriate (37). Clinicians can also refer the patient and their caregiver(s) to social services for further support, as well as help connect them with reliable sources of information and even local research opportunities and clinical trials.

One important role for a clinician treating a patient diagnosed with early-stage AD is to closely monitor the patient's disease progression through regular follow-up appointments (e.g., every 6–12 months); clinicians should encourage patients (and the caregiver) to make additional follow-up appointments, especially should symptoms worsen. Routine cognitive and functional assessments (Table 4) should be used to monitor disease progression; these tools can be used to identify unexpected trends, such as rapid decline, which could prompt the need for additional medical evaluation such as blood tests, imaging, or biomarker analyses. Results from such tests could help guide management and/or treatment decisions over the course of the patient's disease.

Non-pharmacologic therapies (e.g., diet and exercise) may be employed for patients with early AD, with the goal to maintain or even improve cognitive function and retain their ability to perform activities of daily living. For patients in the early stages of disease, dietary changes (e.g., following a healthy diet high in green, leafy vegetables, fish, nuts, and berries), physical exercise, and cognitive training have demonstrated small but significant improvements in cognition (36, 91). Nonpharmacologic therapies can have a positive impact on quality of life and are generally safe and inexpensive (36); however, compliance with these non-pharmacologic therapies should be monitored by the clinician. Research suggests that multimodal therapies, such as cognitive stimulation therapy, may also be more effective when used in combination with pharmacologic treatments (91).

Several pharmacologic treatments have received regulatory approval to treat the symptoms of mild to severe AD dementia. Acetylcholinesterase inhibitors (donepezil, rivastigmine, and galantamine) and N-methyl-D-aspartate receptor antagonists (memantine) can be prescribed to patients to temporarily ameliorate the symptoms of AD dementia such as cognitive and functional decline (92, 93, 94, 95, 96). Meta-analyses of donepezil, rivastigmine, and galantamine have shown that patients with mild-to-moderate AD dementia experience some benefits in cognitive function, activities of daily living, and clinician-rated global clinical state (93, 94, 97). Furthermore, treatment with acetylcholinesterase inhibitors and/or memantine has also been shown to modestly improve measures of global function and temporarily stabilize measures of activities of daily living (96). However, it is important to note that these drugs provide only temporary, symptomatic benefit and that not all patients respond to treatment (36, 98). Critically, none of the current drugs available address the underlying pathophysiology or alter the ultimate disease course.

Following AD diagnosis, a comprehensive approach toward clinical care can be individualized based on the patient's specific AD risk factors (20, 21). Clinicians should consider managing uncontrolled vascular risk factors (e.g., hypertension, hyperlipidemia, diabetes) with antithrombotics, antihypertensives, lipid-lowering, and/or antidiabetic agents, respectively, to reduce the risk of cerebrovascular ischemia and stroke, and subsequent cognitive decline (36, 99). They should also consider the management of the patient's behavioral symptoms. For most patients in the early stages of disease, behavioral symptoms will be relatively mild, and no pharmacologic management is required; however, pharmacologic treatment, such as a low-dose selective serotonin reuptake inhibitor, can be prescribed for patients with AD-associated depression and anxiety (100, 101).

### Specialist clinician checklist

The specialist's role is critical to further evaluating the initial checks/assessments, providing the diagnosis, and developing the individualized patient management plan:

• Identify deficits to specific cognitive domains using appropriate tests
• Confirm functional performance, using patient and caregiver assessments
• Perform structural imaging to complete assessment of the patient
• Confirm diagnosis with imaging or fluid biomarkers
• Develop a personalized management and follow-up plan
• Direct the patient to additional support resources such as the Alzheimer's Association

### Case study: Treat

Following diagnosis, J.K. was advised on the available management options and research opportunities (Table 1E). The specialist emphasized the need to control his vascular risk factors and suggested lifestyle modifications to optimize the management of his other medical problems. The patient's neuropsychiatric symptoms were considered mild and did not require pharmacologic intervention. The patient was also provided with details for a local social worker and directed toward further disease-specific information from the Alzheimer's Association related to his disease. The patient was encouraged to return for additional follow-up visits so that his disease and associated symptoms could be appropriately monitored and managed.

## Future perspectives

An early diagnosis of AD will become increasingly important as treatments that alter the underlying disease pathology become available—particularly given the expectation that such treatments will be more effective in preserving cognitive function, and thus prolonging independence, when given early in the course of the disease (19). The approval of such treatments will likely lead to an increased awareness of cognitive impairment and other AD-associated symptoms among both the public and non-specialists, such as those in primary care settings. This may encourage more patients/family members to seek help at an earlier stage of disease than is currently seen in community practice. Increased use of sensitive screening measures to proactively assess for the presence of AD symptoms will help identify patients suspected of having early AD. Assessment of cognitive impairment during a Medicare Annual Wellness Visit is inconsistent; the U.S. Preventative Services Task Force, whilst recognizing the importance of MCI, has maintained its decision that there is insufficient evidence to support the mandate of cognitive screening. However, sensitive screening procedures, along with the availability of disease-modifying treatments, are likely to change their recommendations. There is also a need for a mandated, standardized screening approach internationally. Together, this will result in an increase in patients requiring diagnosis, increasing the demand for specialists to evaluate and diagnose, the need for amyloid confirmation, and wait times for patients, which will collectively put further pressure on an already-stretched healthcare infrastructure (25).

Nevertheless, efforts continue within the AD field to streamline the diagnostic process. Planning for and implementing change will not only improve patient management now but also help prepare healthcare systems for an approved disease-modifying treatment for AD. A flexible, multidisciplinary team approach is recommended to integrate the care needed to detect, assess, differentiate, diagnose, treat, and monitor a diverse AD population (24). The development of tests that could be carried out routinely in a primary care setting, such as blood-based AD biomarkers, would help PCPs and non-specialists identify which patients may need further evaluation or referral to a specialist (25). Interest also remains high in advancing imaging techniques, such as amyloid and tau PET, to support a diagnosis of AD. Although amyloid and tau PET are not currently readily available, they may be useful for specialists in the future to determine disease staging or track progression, or as a surrogate marker of cognitive status (74). The introduction of new screening and diagnostic tools could ultimately help lower the burden on specialists and ensure patients are diagnosed in a timely manner.

## Conclusions

Consensus within the AD community has recently shifted to encourage the diagnosis of AD as early as possible. This shift will enable patients to plan their future and consider symptomatic therapies and lifestyle changes that could reduce cognitive deficits and ultimately help preserve their quality of life. Promisingly, new, potentially disease-modifying therapeutic candidates are on the horizon that could be effective in early AD by targeting and ameliorating the underlying biological mechanisms (92, 102). This paper has outlined a menu of practical tools for clinicians to use in the real world to support an early diagnosis of AD and how they may best be incorporated into current clinical practice. Ultimately, a coordinated, multidisciplinary approach that encompasses primary care and specialist expertise is required to ensure timely detection, assessment and differentiation, diagnosis, and management of patients with AD.

## Electronic supplementary material

*Electronic supplementary material:* Practical Guidance extender video.

Supplementary material, approximately 65.7 MB.

*Authors' contributions:* All authors participated in the review of the literature and in the drafting and reviewing of the manuscript. All authors read and approved the final version of the manuscript for submission.

*Funding:* The authors developed this manuscript concept during an assessment of Alzheimer's disease educational needs. The development of this manuscript was funded by Biogen. Editorial support was provided by Jodie Penney, MSc, PhD, Helios Medical Communications, Cheshire, UK, which was funded by Biogen.

*Conflict of Interest:* AP reports personal fees from Acadia Pharmaceuticals, Alzheon, Avanir, Biogen, Cadent Therapeutics, Eisai, Functional Neuromodulation, MapLight Therapeutics, Premier Healthcare Solutions, Sunovion, and Syneos; grants from Alector, Athira, Avanir, Biogen, Biohaven, Eisai, Eli Lilly, Genentech/Roche, and Vaccinex. RI has nothing to disclose. MS reports personal fees from Alzheon, Athira, Biogen, Cortexyme, Danone, Neurotrope, Regeneron, Roche-Genentech, and Stage 2 Innovations; stock options from Brain Health Inc, NeuroReserve, NeuroTau, Neurotrope, Optimal Cognitive Health Company, uMethod Health, and Versanum Inc. Additionally, he has intellectual property rights with Harper Collins. SK and IR report employment with Biogen.
